# The Influence of Sedimentation on the Composition of the Lipoaspirate and the Effects on Further Mechanical Processing

**DOI:** 10.3390/cells14080601

**Published:** 2025-04-16

**Authors:** Andreas Eigenberger, Oliver Felthaus, Alexander Bartsch, Tom Schimanski, Kirsten Utpatel, Lukas Prantl

**Affiliations:** 1 Department of Plastic, Hand and Reconstructive Surgery, University Hospital Regensburg, 93053 Regensburg, Germany; 2 Medical Device Lab, Regensburg Center of Biomedical Engineering (RCBE), Faculty of Mechanical Engineering, Ostbayerische Technische Hochschule Regensburg, 93053 Regensburg, Germany; 3 Institute of Pathology, University of Regensburg, 93053 Regensburg, Germany

**Keywords:** autologous fat transfer, lipofilling, intersyringe processing, mechanical processing, fat graft, cell-enriched lipotransfer

## Abstract

Manual processing of lipoaspirate can enhance stem cell concentration, thereby improving the take rate, which still represents a major challenge in autologous fat transfer. However, since the preparation consists of many manual steps that are difficult to standardize, we investigated the influence of residual tumescent solution on the macroscopic and microscopic outcome of the mechanically processed lipoaspirate. Additionally, we investigated whether sedimentation followed by vacuum filtration of the aqueous phase could accelerate processing by replacing the initial centrifugation step. Samples with more than 5% remaining aqueous phase show no clearly defined oil phase, preventing any volume reduction. In contrast, all centrifuged samples produced a clear oil phase. The remaining tissue, as confirmed by both histology and viability assays, was superior to nanofat. Although sedimentation and filtration in the LipoCollector did not sufficiently separate enough aqueous phase from the lipoaspirate, tissue viability was significantly higher compared to our control container. Our findings indicate that centrifugation remains essential for effective aqueous phase separation and further mechanical processing, while the automatic filtration may enhance processing efficiency. These results indicate that further work is needed to simplify mechanical processing, as the outcome can be significantly influenced by parameters such as tumescent impurities.

## 1. Introduction

Autologous fat transfer is no longer a procedure solely established for the treatment of volume deficits or scars in plastic and aesthetic surgery. For instance, it is increasingly being employed in orthopedics to address arthrosis, in otolaryngology to treat dysphonias, and interdisciplinarily in the management of chronic wounds or scars [1,2,3,4,5,6]. However, regardless of the application, the major challenges remain, namely poor reproducibility and sometimes unsatisfactory outcomes [7,8,9]. In some cases, volume losses of up to 70% have even been reported in the literature [10,11]. This is partly due to the fact that no uniform standard for suction and reprocessing has been established. The recent systematic review by McSweeney et al. demonstrates that mechanical fat processing, regardless of whether it involves fragmentation or disruption, enhances vasculogenic potential by increasing levels of angiogenic growth factors and relevant vascular progenitor cells [12].

To improve this situation, the simplest method is a gentle liposuction and minimal traumatic processing to remove impurities before the reinjection. While minimal processing ensures tissue viability is preserved, it does not lead to a significant improvement in graft quality [13]. Alternatively, lipoaspirate can be enzymatically or mechanically processed, resulting in an enrichment of the progenitor cells in the graft [14]. This is important because a higher stem cell concentration correlates with improved graft survival, as already shown by Kolle et al. and Ma et al. [15,16]. Enzymatic digestion, while highly standardizable, is time-consuming, costly, and is considered a substantial alteration of the tissue [17]. On the other hand, mechanical processing methods are more challenging to standardize as the forces applied are mostly dependent on the operator. However, mechanical processing can be performed intraoperatively and is cost- and time-efficient [18,19]. Some of these mechanical processing protocols are mainly designed to reduce the particle size of the tissue so that it can be injected through 21G cannulas. This processed tissue is then referred to as nanofat [20].

If the mechanical forces are high enough that, along with particle size reduction, there is a disruption of mechanically less stable structures, depot adipocytes are broken up, resulting in the release of stored lipids. The stromal vascular cells (SVCs) remain structurally intact in the extracellular matrix (ECM). Interestingly, histological examinations revealed that the mechanically unstable structures primarily consist of adipocyte depots. Smaller adipocytes, which are on average half the size of those in the depots (150 µm) and are more embedded in the ECM, seem to withstand the processing without damage. Consequently, after the removal of the released lipid phase, the cells occupy a reduced volume compared to their pre-processing state, leading to an increased concentration of the SVCs [21]. For better differentiation, the technique is referred to as Cell-Enriched Lipotransfer [22,23]. If this protocol is completely executed, a Cleaned and Enriched LipidTissue–purified long-lasting ultra-concentrated supergraft is obtained (CELT^PLUS^). The key difference from nanofat samples is that after discarding the pure oily phase, the aforementioned volume reduction can occur. Since no oily phase can be identified and discarded after the nanofat processing, no cell enrichment can take place due to volume loss.

Regardless of the chosen fat processing protocol, it is scientific consensus that impurities, such as blood and tumescent solution, should be removed from the lipoaspirate before injection [24,25]. If tissue is to be processed mechanically, the aqueous phase must be separated before subsequent processing steps, as these impurities may impact the outcome of the mechanical processing. However, the rupture of the adipocyte depots and the accompanying enrichment only seems to work if centrifugation has been performed beforehand. Since centrifugation is an increased workload for the operating personnel, there is a need for medical devices to assist in the sedimentation process. As there is less of a sharp phase boundary between the fat-tissue and the impurity, remixing often occurs when the aqueous phase is drained after sedimentation. This could be prevented by using certain containers that allow the aqueous phase to be automatically filtered through suction before transferring the fat tissue into syringes.

In this study, we aim to investigate the influence of residual tumescent solution on the macroscopic and microscopic outcome of the mechanical processing of lipoaspirate for enrichment with ECM and progenitor cells. Since this would represent a significant advantage over labor-intensive centrifugation, we examine whether simple sedimentation followed by phase separation using a sieve in a collector is sufficient to remove enough fluid from the lipoaspirate to obtain CELT^PLUS^, or whether centrifugation remains necessary.

## 2. Materials and Methods

### 2.1. Patient Collective

Four female and one male patient with an average age of 54 years (from 44 to 62) were included in the study. The BMI of the participants at the time of surgery was 30.1 ± 3.5 kg/m^2^. Only patients who had a planned liposuction at Caritas Hospital St. Josef and agreed to participate in our study were included. Patients with a malignant underlying disease, a diagnosed (not yet healed) bloodborne infection, or an acute infection in the donor tissue were excluded. This study was approved by the Ethics Committee of the University Hospital of Regensburg (08/117).

### 2.2. Liposuction Technique

The liposuctions were performed by the University Center for Plastic, Aesthetic, Hand, and Reconstructive Surgery at the Caritas Hospital St. Josef in Regensburg. All patients had general anesthesia. After standardized positioning and disinfection, stab incision was performed and the same volume of warm 0.9% saline (including 1:200,000 epinephrine) was injected as the amount of fat tissue intended for extraction in this area. Both the injection and the subsequent aspiration were performed using the body-jet^®^ evo (Human Med AG, Schwerin, Germany). Following the exposure time of 15 min, a suction cannula (Human Med) with a diameter between 3.8 and 4.4 mm was connected. During liposuction, tumescent solution was continuously applied to the harvesting sites (water jet-assisted liposuction). The aspiration settings on the body-jet^®^ evo were set to the highest possible level in the LipoCollection mode. This ensured that the suction pressure did not drop below 0.5 mbar. The infiltration settings were pulsation short and range 2.

**Figure 1:**
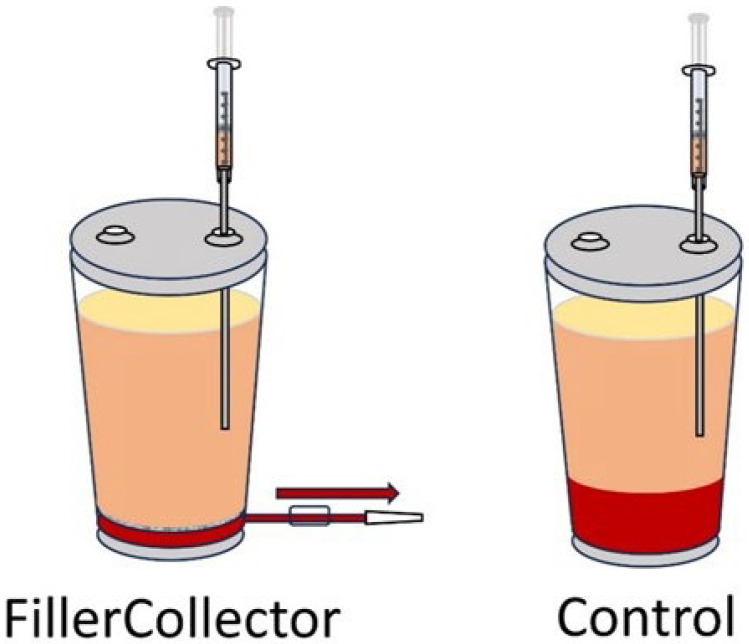
Graphical comparison of the FillerCollector^®^ with a filter above the outlet and our control container in which the aqueous phase could not be discarded through a filter before removal of the adipose tissue.

During aspiration, the lipoaspirate was alternately directed into a container that allows the aqueous phase to be removed from the bottom before the fat extraction (FillerCollector^®^; Human Med AG) or a container without a filter and an additional outlet (Figure 1).

After the last aspiration, a period of 10 min elapsed before the sedimented aqueous phase was drawn through the filter at the lower outlet into an empty suction bag using the body-jet^®^ evo. This suction bag, along with the lipoaspirate remaining in the FillerCollector^®^ and the control container, was transported to the Laboratory for Plastic, Hand, and Reconstructive Surgery at the University Hospital Regensburg for further experiments.

### 2.3. Mechanical Sample Processing

For the mechanical processing, both a 10 mL and a 20 mL Luer Lock syringe were filled from each of the two containers. The 20 mL syringes were processed exactly according to the Cell Enriched LipoTransfer protocol, which has already been described in detail [22]. First, the lipoaspirate was centrifuged at 1600 rcf for 2 min (Rotina 380R; Andreas Hettich GmbH & Co. KG, Tuttlingen, Germany). Then, the aqueous phase was discarded, before the remaining adipose tissue was manually passed 10 times through a 3-way stopcock. In the context of this study, the tissue was always processed by the same experienced operator. As a quality indicator, the sound of turbulent flow was used to confirm that sufficient force was applied during the intersyringe processing.

**Figure 2:**
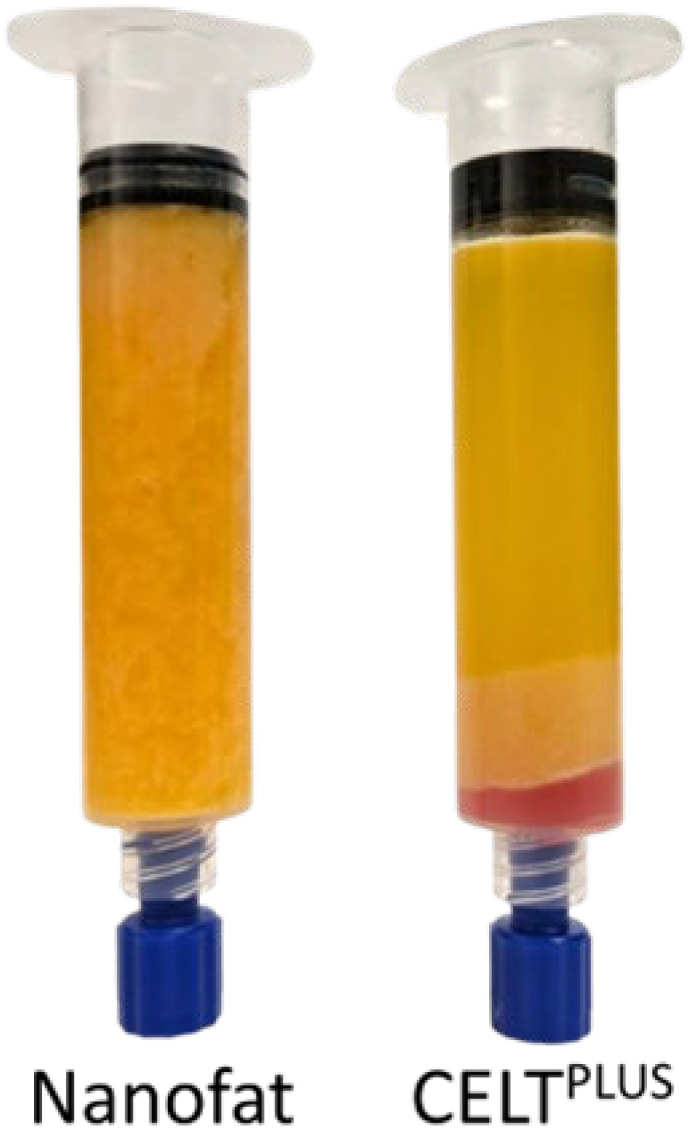
Comparison of the macroscopic appearance of nanofat and CELT after intersyringe processing and the subsequent second centrifugation shows a homogeneous nanofat sample and a clearly separated oil phase with a reduced remaining fat tissue for CELT^PLUS^.

Due to the smaller diameter, the intersyringe processing was always performed with 10 mL syringes, while the first centrifugation step was carried out with 20 mL syringes due to the larger volume. For the directly obtained 10 mL syringes, the first centrifugation step was omitted, and the intersyringe processing was carried out directly. For all samples, the processed tissue was again centrifuged at the same settings. The Cell-Enriched Lipotransfer protocol was successful if a distinct oily phase was seen above the remaining tissue after the second centrifugation so that the volume reduction of the tissue can lead to an accumulation of ECM and SVF (CELT^PLUS^). If there is only a reduction in the particle size but no oily phase after the second centrifugation, the processing of the tissue resulted in nanofat (Figure 2).

To determine the proportion of aqueous impurities in the lipoaspirate that results in no clearly separated oil phase after complete processing, we conducted an additional test. In this test, all impurities released from the lipoaspirate were discarded after the first centrifugation. Then, 5, 10, or 15% *v*/*v* of tumescent solution was added (n = 4). We ensured the tumescent solution was homogeneously distributed in lipoaspirate before processing the fat as previously described and analyzing the results.

### 2.4. Macroscopic Sample Composition

The remaining tissue of the FillerCollector^®^ was transferred into 20 mL syringes and centrifuged at 1600 rcf for 2 min (Rotina 380R; Andreas Hettich GmbH & Co. KG). To quantify the aqueous phase separated during centrifugation, each syringe was photographed under standardized conditions. Subsequently, the height of the aqueous phase and the fat phase were measured along the centrifugation gradient using ImageJ 1.53e (U.S. National Institutes of Health, Bethesda, MD, USA). Each syringe was measured three times, one time in the center and two times lateral.

### 2.5. Viability Assays

The tissue viability of sedimented as well as centrifuged adipose tissue from both containers were investigated. Additionally, tissue particles discarded by the FillerCollector^®^ were also investigated using a resazurin assay. For this, 50 µL of adipose tissue per sample were mixed with 400 µL of supplemented cell culture medium (10% FCS, 1% P/S) and 50 µL of 0.7 mM resazurin (Sigma-Aldrich, St. Louis, MO, USA) in a 2 mL Eppendorf cup. The mixture was then incubated for one hour at 37 °C using an overhead shaker. After incubation, samples were gently centrifuged and 100 µL of the aqueous phase were pipetted to a 96-well plate. Fluorescence intensity was measured by the Varioskan Flash (Thermo Fisher Scientific, Waltham, MA, USA) with 530 nm excitation and 590 nm emission wavelengths.

### 2.6. Histologic Sample Preparation and Evaluation

All tissue samples were preserved in a neutral buffered formalin solution and subsequently embedded in paraffin. Thin histological sections, measuring 4 μm in thickness, were meticulously crafted. These sections were then subjected to deparaffinization using a mixture of ethanol and xylene, followed by staining in accordance with our established hematoxylin and eosin standard protocol. Subsequently, all the samples underwent digital scanning using the P1000 scanner (3D Histech Kft., Budapest, Hungary). Qualitative comparison of the histologic images was performed using CaseViewer (3D Histech Kft.).

### 2.7. Statistical Analysis

Due to the limited sample size, we performed an analysis of variance (ANOVA) to compare the mean values of several groups in every experiment. If only two equal-sized groups were compared with each other, we used the Student’s *t*-test. Values of *p* < 0.05 were considered statistically significant and values of *p* < 0.001 were considered highly statistically significant.

## 3. Results

### 3.1. Differences Between CELT^PLUS^ and Nanofat

**Figure 3:**
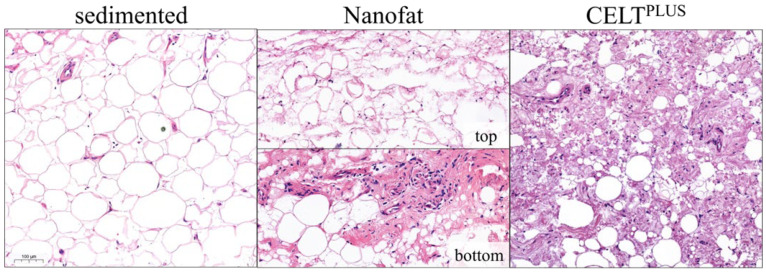
Comparison of the microscopic composition of sedimented lipoaspirate and the processed CELT^PLUS^ and nanofat samples with the distinction between nanofat that accumulates at the bottom or top in the sample after the second centrifugation (HE staining).

The macroscopic and histological compositions of CELT^PLUS^ and nanofat differ substantially. CELT^PLUS^ is compact, homogeneous, and seems to consist mainly of large areas of extracellular matrix in which many cell nuclei were stained. The areas of varying sizes within the ECM that are not stained, and where many cell nuclei can be observed, are most likely to correspond to blood vessels. Furthermore, small adipocytes with a univacuolar lipid droplet can likely be seen. Even though the macroscopic appearance of nanofat is homogeneous, histologically there are significant differences along the centrifugation gradient. At the bottom of the sample, the histological image is similar to the CELT^PLUS^ sample but slightly less compact. Further up the sample, the compactness decreases, and the individual adipocyte- or ECM-fields are widely distributed. These histological slides are more comparable to exclusively sedimented lipoaspirate, although the latter also appears to be denser. In addition, significantly more cell fragments can be observed, which is shown in Figure 3.

**Figure 4:**
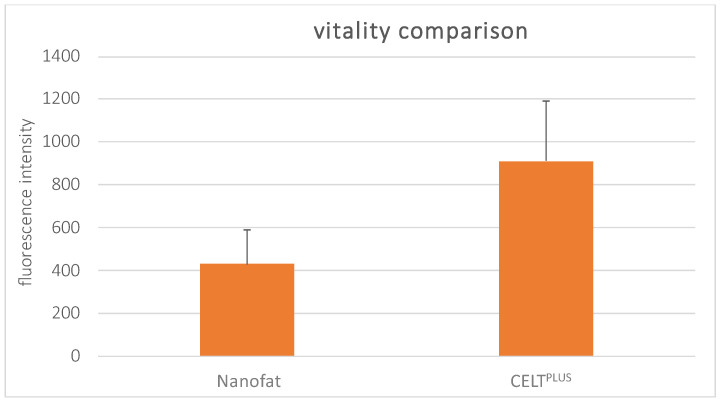
Comparison of fluorescence intensity of nanofat and CELT^PLUS^ samples after the resazurin viability assay.

As shown in Figure 4, the viability assays also revealed significant differences between the CELT^PLUS^ and nanofat samples after complete processing and the removal of the non-viable oily and aqueous phases (*p*-values < 0.05). On average, the fluorescence intensity of the CELT^PLUS^ samples was 2.75 times higher than that of the nanofat samples.

### 3.2. Impact of Remaining Tumescent Solution on the Outcome of Mechanical Processing

In samples where the aqueous phase constituted 10% or more of the volume fraction, a distinctly separable oil phase was never observed after complete processing. Even with only 5% contamination, CELT^PLUS^ could only be achieved in one of four samples. The results are detailed in Table 1.

### 3.3. Preventing a Re-Mixing of Phases After Sedimentation

Since centrifugation is time-consuming and resource-intensive, it was investigated whether extensive sedimentation is sufficient to remove enough of the aqueous phase from the lipoaspirate. Exclusively in one of the five patients, a released lipid phase could be observed with exclusively sedimented adipose tissue from the FillerCollector^®^. The comparable sample from the same patient as well as all other samples from the control container resulted in nanofat. In all samples, whether from the FillerCollector^®^ or the control container, which were centrifuged at the beginning of CELT^PLUS^ processing, a homogeneous oil phase was observed after the complete processing. For a better overview, these results have been listed in the following table (Table 2). The volume of the remaining tissue phase was approximately 1 mL in all patients.

### 3.4. Remaining Aqueous Phase After Sedimentation

**Figure 5:**
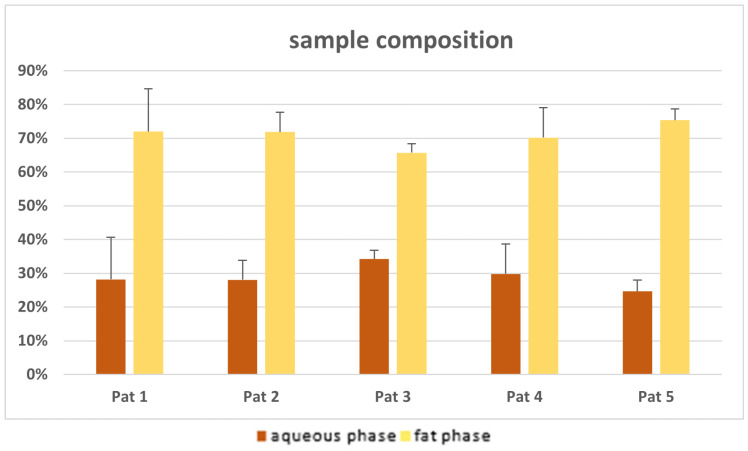
The proportion of the aqueous and fat phases after centrifugation of the lipoaspirate filtered in the FillerCollector^®^.

After centrifugation, the filtered adipose tissue from the FillerCollector^®^ consisted, on average, of 29.1 ± 7.8% aqueous phase. The values for individual patients are compared in the following diagram (Figure 5). The differences shown in the remaining aqueous phase between the five patients are not statistically significant (*p*-values > 0.05). At least eight syringes were measured for each patient, corresponding to a volume of approximately 160 mL.

### 3.5. Comparison of Tissue Viability

**Figure 6:**
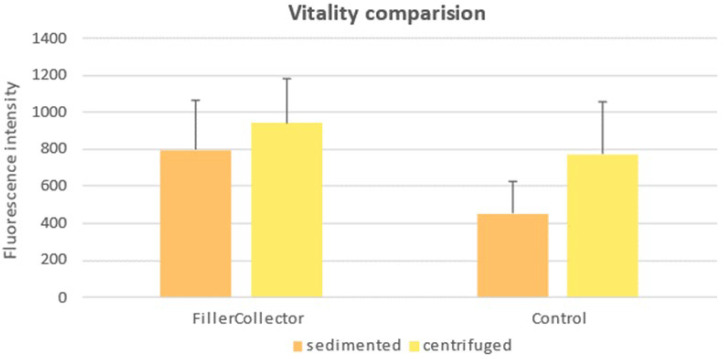
Comparison of the resazurin assay for sedimented and centrifuged tissue from the FillerCollector^®^ and the control container.

The comparison of tissue viability shows that the sedimented as well as the centrifuged tissue from the FillerCollector^®^ display a significantly higher fluorescence intensity than the comparable samples from the control group (sedimented: *p*-value < 0.001; centrifuged: *p* value < 0.05). The mean values of the fluorescence intensities of the individual samples are compared in Figure 6. The centrifugation and discard of the released aqueous phase increased the fluorescence intensity of the lipoaspirate from the FillerCollector^®^ by a factor of 1.2 ± 0.1. For the lipoaspirate from the control container, the fluorescence increased by a factor of 1.7 ± 0.5 due to the centrifugation. The difference in the increase in fluorescence intensity was statistically highly significant (*p*-value < 0.001).

After the first centrifugation, the released aqueous phase was discarded. In two patients, this aqueous phase was examined for the presence of metabolically active cells using resazurin assays. The fluorescence intensity of the aqueous phase was slightly higher than the blanks with mean values of 1.6 ± 1.6 (FillerCollector^®^) and 5.0 ± 1.5 (Control). Compared with the fluorescence intensity of the fat tissue from both containers, it is highly significantly smaller by more than a factor of 150 (both *p*-values < 0.001). For better comparability, the fluorescence intensity values for the aqueous and fat phases from both containers are compared in Table 3.

### 3.6. Analysis of the Filtered Discard

**Figure 7:**
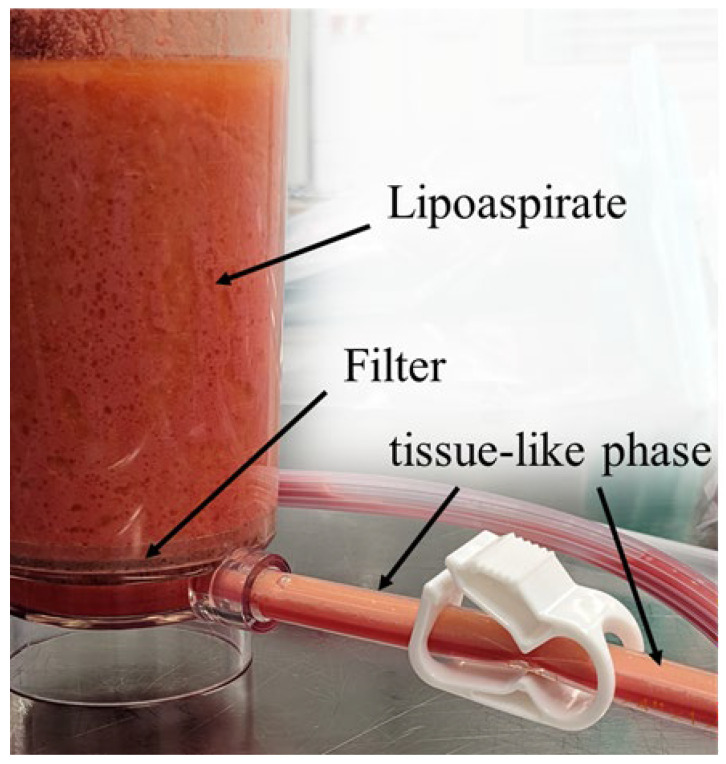
Overview of the FillerCollector^®^ and the stored lipoaspirate; the filter and the outlet for the aqueous phase show the tissue-like phase.

In the suction bag into which the aqueous phase from the FillerCollector^®^ that had been sucked through the filter was collected, a slight tissue-like phase floating on top of the aqueous phase was observed. This filtered tissue-like phase was most clearly observed at the outlet of the FillerCollector^®^ (Figure 7).

**Figure 8:**
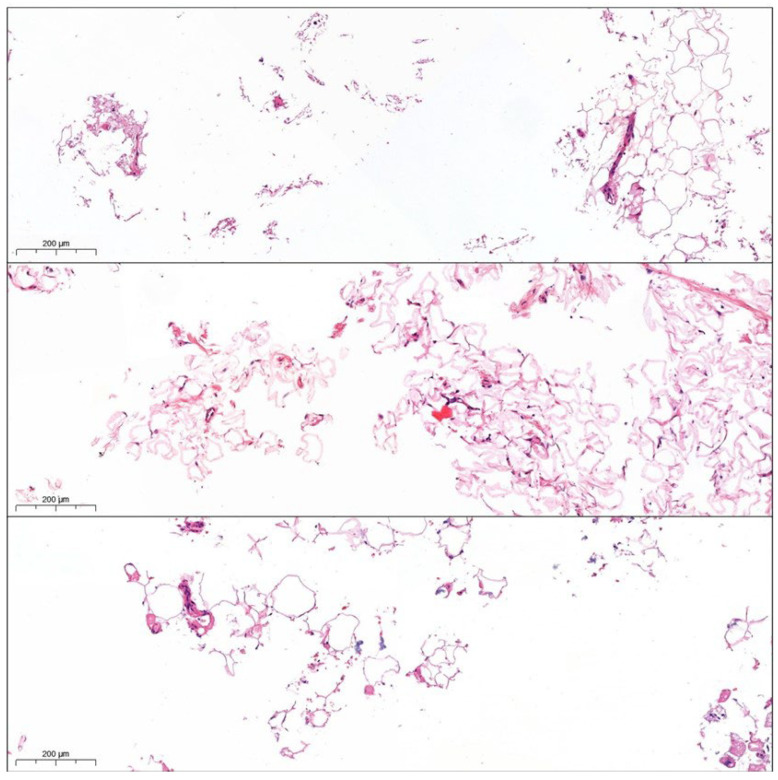
Representative sections of HE-stained histological scans of the tissue-like phase.

The histological preparations show that this phase consists mainly of cell fragments, extracellular matrix, small accumulations of adipocytes, and single adipocytes. However, compared to normal lipoaspirate, these are widely spread on the slide, which is exemplified in Figure 8.

**Figure 9:**
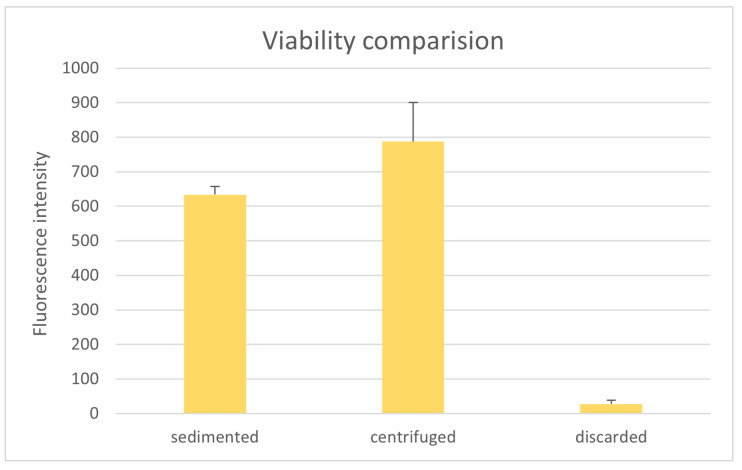
Comparison of the fluorescence intensity of the sedimented, the centrifuged lipoaspirate from the FillerCollector^®^ and the filtered and discarded tissue-like phase.

For one patient, the tissue-like phase was transferred to a syringe; after sedimentation for 5 min the aqueous phase was discarded, and a resazurin test was performed. As shown in Figure 9, the fluorescence intensity of the discarded tissue-like phase is highly significantly smaller than that of the sedimented or centrifuged lipoaspirate from the FillerCollector^®^ (both *p*-values < 0.001). However, it should be noted that this value is 18 times higher than that of the rest of the aqueous phase.

## 4. Discussion

Autologous fat transfer is an interdisciplinarily established procedure used worldwide [26]. Compared to the use of implants, the procedure involves fewer complications. Additionally, once the fat has integrated, it changes naturally with the patient’s weight fluctuations. Due to the difficult reproducibility and sometimes lower engraftment rates, surgeons strive to prepare the best possible graft to avoid an unnecessarily high number of surgeries, for example, to fully correct a volume defect.

Therefore, after a successful liposuction procedure, it is necessary to remove the tumescent solution and impurities resulting from blood from the fat tissue before proceeding with the injection [24,25]. This is not solely relevant due to its effect on further mechanical processing. Aqueous and bloody contaminants in the lipoaspirate can also impair the viability of fat grafts and stem cells [27]. Furthermore, the tumescent solution is rapidly absorbed by the body, leading to unsatisfactory volume loss within a few days.

This necessity is also confirmed by our viability assays, which show that almost no metabolically active cells are present in the aqueous phase. At this point, it should be noted that this result could, in principle, also be attributed to the manipulation of the cells’ metabolic activity. However, this could not be observed in other studies conducted by our research group [23]. It appears that extensive 10 min sedimentation after the liposuction was completed is not sufficient to separate the aqueous phase from the adipose tissue, as the tissue in the FillerCollector^®^ still reproducibly consisted of 29.1% aqueous phase, which was only released after centrifugation. It should be noted that the lipoaspirate, once aspirated, undergoes sedimentation within the FillerCollector during the liposuction. Overall, the sedimentation time we used was comparable with the study by Yin et al. [28].

Presumably due to this yield of remaining tumescent solution in containers, the shear forces during the intersyringe processing are apparently not sufficient to break up the adipose tissue as described by Patel et al. [29]. However, this volume reduction of the adipocyte depots is essential for the enrichment of the stromal vascular cells [21]. It has already been shown that this enrichment leads to satisfactory long-term results for facial lipofilling [30]. From both containers, mechanical processing of almost all samples in which the first centrifugation step was omitted resulted in nanofat without a separated oil phase after the second centrifugation.

Since the flow rate and the connector diameter were the same for all samples, this can only be attributed to the aqueous contamination. Therefore, we can add to the findings of Osinga et al., Tonnard et al., and Banyard et al. that the result of intersyringe processing does not only corelate with the flow rate; it is also significantly influenced by the composition of the lipoaspirate [20,31,32], as aqueous impurities of more than 5% *v*/*v* already lead to the production of nanofat only. However, the present results show that even if the filter above the outlet is used to prevent the impurities from mixing with the fatty tissue again when draining, the centrifugation alone does not remove enough aqueous phase from the tissue for the desired mechanical enrichment. Therefore, centrifugation cannot be omitted.

In contrast to the processing parameters of Osinga et al., those used here lead to a significant change in the histological composition of the tissue [31]. Regardless of whether the resulting tissue preparation is referred to as nanofat or CELT^PLUS^, a few residual adipocytes could still be observed after mechanical processing. This has already been described by the working group of Girard et al. and represents a clear difference to enzymatic digestion [33]. Although the lower phase of the nanofat is comparable to the histological composition of the CELT^PLUS^ tissue, it appears to be less dense. The border to the nanofat phase with significantly fewer stromal vascular cells and many adipocyte fragments appears to be smooth. It should be noted that the term nanofat is not used consistently in the literature and may also describe a tissue preparation that leads to an increase in stromal vascular cells per volume [20,34]. However, in this paper, for better distinction, adipose tissue in which no volume loss was observed was referred to as nanofat, and adipose tissue in which a significant volume loss was observed was referred to as CELT^PLUS^. The differences between the already established mechanical treatments should be investigated in a prospective study. However, it should be noted that Tran et al. had already shown that the transplantation of nanofat leads to oil cysts [35]. In addition, this can lead to lipid toxicity and subsequent tissue inflammation [36,37].

Even though the FillerCollector^®^ made no difference for the outcome of the Cell Enriched LipoTransfer protocol, the resazurin assay shows that the tissue viability of the tissue in the FillerCollector^®^ was significantly higher than in the control container. This could indicate a lower number of metabolically active cells in the fat tissue of the control container [38]. After centrifugation, the viability difference between the two containers becomes significantly smaller than the viability difference between the sedimented samples (see Figure 6). This may be due to the fact that the fat and aqueous phases recombine while the tissue is removed from the control container.

One disadvantage of the FillerCollector^®^ seems to be that when the aqueous phase is drained through the filter, a tissue-like phase is also discarded. This has already been observed in comparable systems [39]. Even if this is only a small volume compared to the fat in the LipoCollector^®^, especially in patients with small fat deposits, one cannot afford to lose viable adipose tissue. However, we were able to show that this tissue-like phase consists almost exclusively of cell fragments and contains only a few metabolically active cells, which is why this should be negligible.

Besides the ability to remove excess tumescent fluid during the liposuction, the greatest advantage of the FillerCollector^®^ appears to be that it prevents the sedimented phases from mixing again. This could not only be helpful for protocols with sedimentation, but it can also significantly increase the efficiency of the centrifugation and thus reduce the workload. Both could lead to more surgeons using centrifugation in the future than the 34% described by Kling et al. [40], which might lead to more reproducible and better take rates [41,42].

As a limitation of this study, it should be noted that the donors tended to have a higher BMI than the average lipofilling patient [43]. However, based on experience, BMI plays a negligible role in the outcome of mechanical processing.

## 5. Conclusions

Although sedimentation and phase filtration can significantly improve the efficiency of the subsequent steps, our results show that the centrifugation step cannot be omitted if the lipoaspirate is to be mechanically processed to obtain CELT^PLUS^. Mechanical processing with excessive residual water in the tissue does not achieve the desired concentration of ECM and SVF and carries the risk of oil cyst formation.

In addition, the automatic removal of the aqueous phase by the filter could minimize the operator-specific influence on the process variation and could, therefore, be useful in a variety of mechanical processing protocols. Further studies should prospectively investigate the different properties of the various tissue preparations to clarify the inconsistent terminology used in the literature and the influence of tumescent solution on the rheological properties of the graft.

## Figures and Tables

**Table 1:** Results of the mechanical processing depending on the amount of tumescent solution in the lipoaspirate. Samples with a distinct oil phase are referred to as CELT^PLUS^; samples with no oil phase are referred to as nanofat.

	Volume Percentage of Aqueous Contamination			
Replicate	0%	5%	10%	15%
1	CELT^PLUS^	CELT^PLUS^	Nanofat	Nanofat
2	CELT^PLUS^	Nanofat	Nanofat	Nanofat
3	CELT^PLUS^	Nanofat	Nanofat	Nanofat
4	CELT^PLUS^	Nanofat	Nanofat	Nanofat

**Table 2:** Results of the mechanical processing for the exclusively sedimented or centrifuged lipoaspirate from the control container and the FillerCollector^®^, where re-mixing of the phases was prevented by removing the sedimented tumescent solution through a sieve at the bottom before extracting the fat.

	FillerCollector^®^		Control	
Patient	Sedimented	Centrifuged	Sedimented	Centrifuged
1	CELT^PLUS^	CELT^PLUS^	Nanofat	CELT^PLUS^
2	Nanofat	CELT^PLUS^	Nanofat	CELT^PLUS^
3	Nanofat	CELT^PLUS^	Nanofat	CELT^PLUS^
4	Nanofat	CELT^PLUS^	Nanofat	CELT^PLUS^
5	Nanofat	CELT^PLUS^	Nanofat	CELT^PLUS^

**Table 3:** Comparison of the fluorescence intensity for the aqueous phase discharged after the first centrifugation and the remaining fat phase from both containers.

Fluorescence Intensity	FillerCollector^®^	Control
aqueous phase	1.6 ± 1.6	5.0 ± 1.5
fat phase	1057.7 ± 290.9	772.7 ± 203.5

## Data Availability

Data are contained within the article.
